# Polyelectrolyte Gels Formed by Filamentous Biopolymers: Dependence of Crosslinking Efficiency on the Chemical Softness of Divalent Cations

**DOI:** 10.3390/gels7020041

**Published:** 2021-04-08

**Authors:** Katrina Cruz, Yu-Hsiu Wang, Shaina A. Oake, Paul A. Janmey

**Affiliations:** 1 Department of Physiology, Institute for Medicine and Engineering, University of Pennsylvania, Philadelphia, PA 19063, USA; kncruz@wustl.edu (K.C.); yuewang@UTMB.EDU (Y.-H.W.); shainaoake@gmail.com (S.A.O.); 2 Department Biochemistry and Molecular Biology, University of Texas Medical Branch, Rm 6.160, 11th and Mechanic St., Medical Research Building, Galveston, TX 77555, USA

**Keywords:** vimentin, Pf1 virus, counterion, rheology

## Abstract

Filamentous anionic polyelectrolytes are common in biological materials. Some examples are the cytoskeletal filaments that assemble into networks and bundled structures to give the cell mechanical resistance and that act as surfaces on which enzymes and other molecules can dock. Some viruses, especially bacteriophages are also long thin polyelectrolytes, and their bending stiffness is similar to those of the intermediate filament class of cytoskeletal polymers. These relatively stiff, thin, and long polyelectrolytes have charge densities similar to those of more flexible polyelectrolytes such as DNA, hyaluronic acid, and polyacrylates, and they can form interpenetrating networks and viscoelastic gels at volume fractions far below those at which more flexible polymers form hydrogels. In this report, we examine how different types of divalent and multivalent counterions interact with two biochemically different but physically similar filamentous polyelectrolytes: Pf1 virus and vimentin intermediate filaments (VIF). Different divalent cations aggregate both polyelectrolytes similarly, but transition metal ions are more efficient than alkaline earth ions and their efficiency increases with increasing atomic weight. Comparison of these two different types of polyelectrolyte filaments enables identification of general effects of counterions with polyelectrolytes and can identify cases where the interaction of the counterions and the filaments exhibits stronger and more specific interactions than those of counterion condensation.

## 1. Introduction

There are many kinds of polyelectrolyte gels. They can be made from flexible polymers like polyacrylates, semiflexible polymers like DNA, or rodlike biopolymers like microtubules. They can be crosslinked by covalent, or noncovalent bonds. Among the most interesting biologically, and least understood chemically, are gels made from the semiflexible and rodlike filaments that make up the cytoskeleton, and similar filamentous polyelectrolytes such as some types of bacteriophages that are present at sites of infection. These highly elongated filaments, which are typically 10s of nanometers in diameter and several microns long can have a surface charge density similar that to that of double stranded DNA [1], interact with counterions by multiple mechanisms and form a variety of interlinked filament systems.

Perhaps the most studied cytoskeletal polyelectrolyte is F-actin, the polymer of actin that forms filaments of 8 nm diameter and a persistence length of 10 µm. Tang et al. showed that F-actin interacts with multivalent cations in a manner roughly similar to the well documented effect of multivalent cations to condense DNA, but with the difference that the much higher stiffness of F-actin also leads to formation of nematic and other liquid crystalline phases that exhibit a complex interplay with filament bundling driven by counterion condensation [2,3]. Divalent cations not only bundle F-actin, but at concentrations lower than those needed for bundling, they increase the elastic moduli of actin networks. Working with semi-dilute solutions of actin filaments (5.8 µM actin = 0.24 mg/mL) studies using passive microrheology [4] find that the shear modulus of actin networks rises an order of magnitude as [Mg^2+^] is raised from 2 mM, a condition at which filaments interact largely sterically, to 52 mM, which begins to induce bundling. Intermediate concentrations provide crosslinks between filaments that increase the shear modulus at small strains but are labile at long times or higher forces.

Similar studies showed that other cytoskeletal polymer like microtubules and intermediate filaments could also be condensed by multivalent cations [5], suggesting that such effects might be generally important in both the normal structuring of the cytoskeleton and the pathological aggregation of the filaments when released into the extracellular space [6], or when the ionic composition of the cell becomes dysregulated [7,8]. 

**Figure 1:**
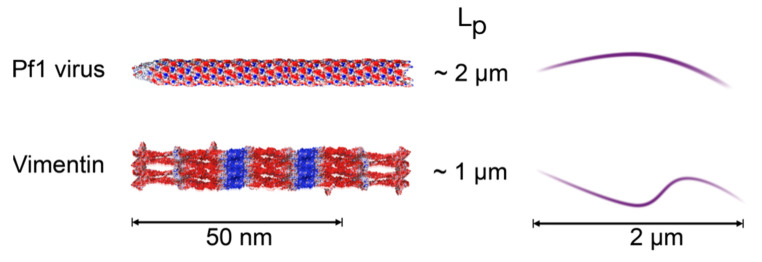
Schematic diagram of the polyelectrolyte filaments Pf1 virus and vimentin intermediate filaments (VIF). Electrostatic potentials are adapted from ref [1]. Red is negative, and blue is positive. Sketches on the right represent the configuration of 2 µm filaments with the persistence lengths (L_P_) of these polyelectrolytes.

Here we focus on two types of filamentous biopolymer, one representing a class of cytoskeletal filaments called intermediate filaments, and the other a filamentous bacteriophage called Pf1. These two classes of biopolymer are biologically unrelated and structurally highly dissimilar, but they share critical features of physical chemistry. Intermediate filaments formed by the protein vimentin contain multiple alpha helical coiled coil proteins interlinked to make a polymer with negative charge density on its surface due to the excess in acidic amino acids over basic amino acids in the polypeptide structure [9]. Pf1 bacteriophages in contrast are formed by a single loop of DNA that is coated with peptides characterized by a positively charged sequence that interacts with the DNA, followed by a hydrophobic surface that forms the water impenetrable barrier protecting the DNA, and ending with an acidic sequence that renders the bacteriophage hydrophilic and imparts on it a negative surface charge density [10]. Vimentin intermediate filaments (VIFs) and Pf1 have very similar surface charge densities of approximately 0.5 negative charges per square nanometer, similar to the surface charge density of double stranded DNA; they have similar diameters of approximately 10 nanometers, and they have similar persistence lengths of approximately 1 micron. In vitro vimentin polymerizes into a polydisperse length distribution of filaments, but Pf1 virus forms an almost perfectly monodisperse solution of filaments with a length of 1.8 microns. The structures of VIFs and Pf1 are schematically shown in Figure 1.

These two structurally diverse but physically similar polymers provide a useful test system to study how divalent and multivalent counterions interact with these charged filaments, and to identify generic and chemically specific effects of divalent metal ions or polyvalent organic cations on network formation and bundle formation by these two types of polymers. The results highlight a remarkably large and apparently systematic difference in the effectiveness by which different metal ions cause these two types of anionic filaments to form bundles, with divalent transition metal ions being much more effective than alkaline earth metals in bundling the polymers, and with the effectiveness of the transition metals increasing with increasing atomic number. Differences in the way that counterions interact with semi flexible polymers can cause them to assemble into networks and bundles point to potentially important interactions that can occur in physiological or pathophysiological conditions. 

## 2. Results and Discussion

### 2.1. Rheology of Gels Formed by Pf1 and Divalent Cations

**Figure 2:**
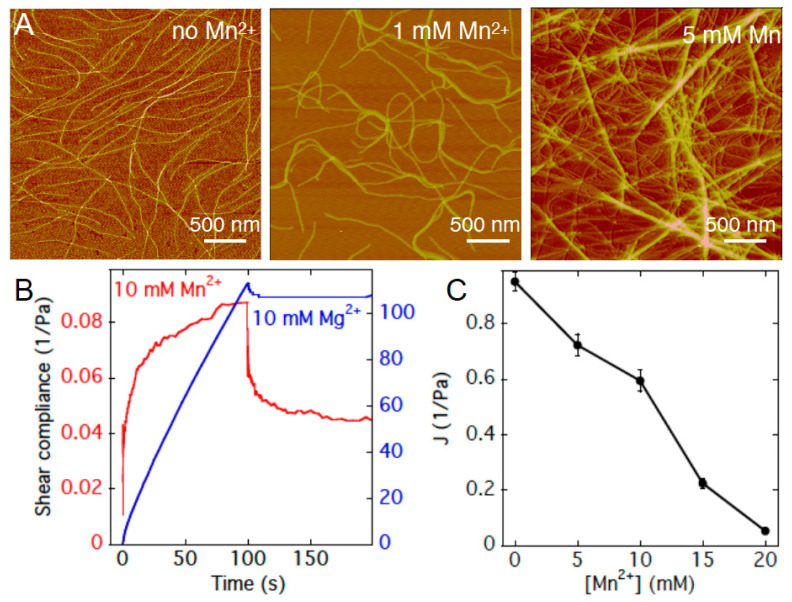
Gelation of Pf1 virus by Mn^2+^. (**A**). Atomic force micrograph of Pf1 virus in suspensions of variable amounts of Mn^2+^. (**B**). Creep and recovery of Pf1 suspensions with 10 mM Mg^2+^ or Mn^2+^ after imposition of a constant shear stress. (**C**). Compliance at 10 s of Pf1 suspensions with various concentrations of Mn^2+^. Error bars denote standard errors of separate experiments imposing 0.04 to 0.2 Pa shear stress.

**Figure 3:**
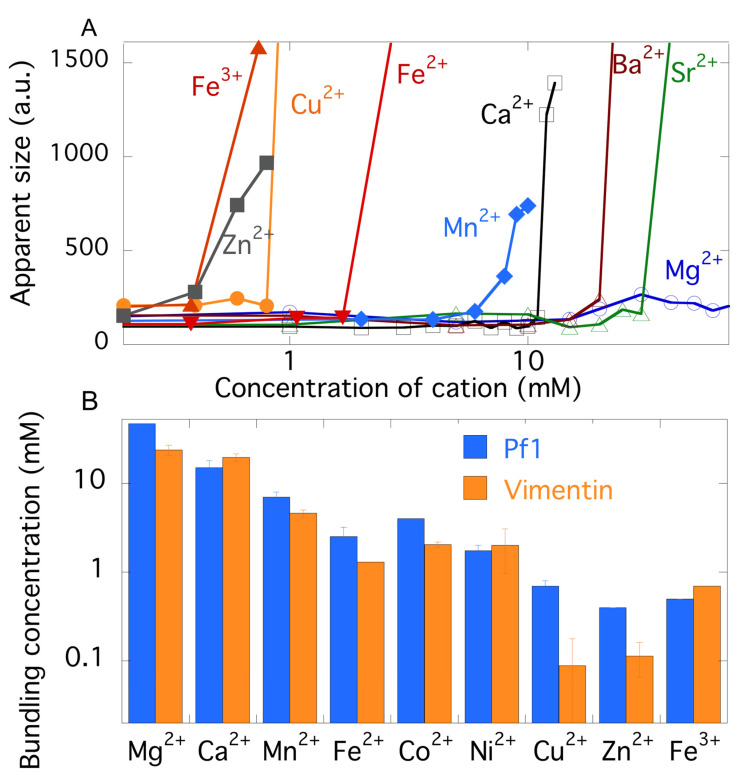
Counterion dependent attraction of like charged polyelectrolytes by various divalent and higher valence cations. (**A**). Changes in apparent size calculated from the diffusion constant as measured by decay of light scattering autocorrelation function for initial suspensions of single virus as they are titrated with increasing concentrations of biologically relevant alkaline earth and transition metal ions. (**B**). Comparison of the efficiency of different metal counterions to aggregate Pf1 and vimentin intermediate filaments (VIFs). Error bars denote standard errors for experiments in which 3 separate preparations of Pf1 or VIFs were titrated by the counterions. In all cases the correlation functions were averages of at least five different measurements of the same sample.

Previous studies showed that Pf1 suspensions at concentrations below their nematic transition form isotropic viscoelastic gels after addition of divalent metal counterions. The efficiency of gelation depends on the type of metal ion. Higher concentrations of the same divalent ions cause the system to phase separate into bundles, with a resulting loss of gel elastic properties when the bundles are too sparse to interpenetrate [11]. Figure 2A shows how Pf1 filaments are progressively assembled into loops and interdigitating bundles. Atomic force micrographs show single rodlike Pf1 virus in the absence of Mn^2+^; with 1 mM Mn^2+^ loops of Pf1 filaments appear as do sites where two viruses attach and branch. These concentrations are below those at which the system phase separates into large, isolated bundles and a very dilute phase as shown in ref. [11] and Figure 3A. At 10 mM Mn^2+^, there appear larger bundles of Pf1 that interdigitate with each other, consistent with the formation of network structures that can resist shear stresses. 

Further characterization of the cation-specific gelation of Pf1 virus suspension is shown in Figure 2B, where the shear creep in response to a constant stress and the resulting recovery after removal of stress are shown. In 10 mM Mn^2+^, Pf1 viruses form a viscoelastic material with very low shear compliance that reaches a steady level of strain at constant stress. After removal of stress, the sample recovers much of its deformation, but exhibits some plastic deformation, as expected if the crosslinks formed by the counterions are labile and can reform in the strained state [11]. In contrast, Pf1 suspensions with the same concentration of Mg^2+^ exhibit very large compliance, with no sign of a steady plateau in strain after 100 s, and nearly no recovery after the stress is released. The difference in the effects of Mg^2+^ and Mn^2+^ on Pf1 is an interesting contrast with the fact that these ions have similar effects on DNA, a much more flexible polymer with a similar surface charge density [12]. This contrast might be related to the effects of polyelectrolyte flexibility on the potentials of mean force (PMFs) between the polyelectrolytes (PEs). Monte Carlo simulations of the PMFs between like-charged PEs with different bending flexibility and a cylindrical confinement of counterions around each PE show that, at least with trivalent counterions, attractive PMFs can be weakened by bending flexibility [13].

### 2.2. Divalent Counterion Specificity in Promoting Attractions between Like-Charged Filaments

Differences in the efficiency of different divalent cations to promote attractive interactions between Pf1 virus are more thoroughly documented in Figure 3. Rheological measurements of gelation require too much material to feasibly survey a large number of conditions, but dynamic light scattering provides a sensitive measure of the onset of attractive interactions between filaments as judged by the decrease in the decay rate of autocorrelation functions of the light scattering fluctuations, equivalent to an increase in apparent particle size, as single filaments begin to interact with each other and move more slowly. These changes in the correlation time occur significantly before the abrupt increase in total scattering intensity that occurs when filaments undergo phase separation into large aggregates. Figure 3A shows a representative experiment in which light scattering is measured from a constant concentration of Pf1 virus with increasing concentrations of four alkaline earth ions and the biologically relevant transition metal ions Mn^2+^, Fe^2+^. Cu^2+^, and Zn^2+^. The abrupt increase in the apparent particle size occurs over a very narrow range of concentrations for three of the four alkaline earth cations, and the critical concentration at which the transition occurs is significantly different for each type of cation. For magnesium, in contrast, the slowdown in filament fluctuation occurs relatively gradually, consistent with its much weaker ability to form strong networks compared to other cations. The order in which the different alkaline earth cations bundle Pf1 does not appear to follow any simple pattern, scaling neither with atomic weight nor with solubility of acetate ions with these metals [14], a quantity that might be expected to be relevant if the divalent cations interact specifically with the carboxyl groups that constitute most of the negative charge of the virus. The greater efficiency of Ca^2+^ compared to Mg^2+^ in forming attractions between Pf1 is the same as that reported earlier for their abilities to bundle the closely related but shorter fd virus [15] and might depend on the greater propensity of Mg^2+^ to remain fully hydrated when it is attracted to negatively charged surfaces [16].

In contrast to the high concentrations of alkaline earth cations needed to bundle Pf1 virus, transition metal ions in the first row of the periodic table are much more efficient and follow a regular trend with increasing efficiency roughly scaling with increased atomic weight of the cation. The experimental conditions have been designed such that the valence of these cations is expected to be +2, and changes in valence, such as the oxidation of ferrous iron to ferric ion can lead to large differences in the ability of iron to bundle Pf1 or VIFs. Ferrous ion is much less efficient than zinc or copper, but ferric ions are more efficient than the divalent cations. In the case of the transition metal ions’ increased efficiency scaling with atomic number, there appears to be a regular pattern, but the underlying mechanism for this effect is not clear. 

### 2.3. Comparison of Counterion-Mediated Attractions in Networks of PF1 and Vimentin

Whether the trend of different divalent ions in bundling Pf1 virus is due to specific interactions with the viral coat or to generic interactions caused by polyelectrolyte effects can be seen by comparison with the efficiency of these ions to bundle vimentin intermediate filaments, which have a similar diameter and charge density as well as flexibility. Figure 3B compares the bundling efficiency of a subset of divalent cations for both Pf1 and VIFs. The similarity in the efficiency of the different metal ions is striking, especially the relatively weak efficiency of alkaline earth ions and the increasing efficiency of transition metals with increasing atomic weight. There are however some exceptions. For example, zinc and copper are significantly more efficient in bundling VIFs than Pf1 virus, and this might reflect the reports that Zn^2+^ can interact with the sulfhydryl group of vimentin [17,18], an amino acid that Pf1 virus lacks and that can also bind Cu^2+^. Indeed copper and zinc have been used to form crosslinked networks of VIFs that increase their elastic modulus well above the levels possible with other divalent cations [19]. The efficiency of Zn^2+^ and Cu^2+^ is greater than that of Fe^3+^, showing that some factor other than valence is responsible for this increased affinity. On the whole however the similarity of the ability of divalent metal ions to bundle the structurally unrelated Pf1 virus and vimentin filaments is an indication of how strong and potentially selective counterion effects can be. These results suggest that under some conditions especially with ions such as zinc and copper, which become dysregulated in some pathological states, cation-mediated attractive interactions between cytoskeletal filaments might be significant [7,20]. This possibility is consistent with the recent report that divalent and trivalent cations such as lead, ferric iron, or aluminum can stiffen cells, as measured by atomic force microscopy, an effect that might be related to the toxicity of these metals [21]. 

The critical concentration at which polyelectrolytes assemble as judged by dynamic light scattering varies with differences in monovalent salt concentration, and excluded volume effects caused by large macromolecules or confinement in small spaces. Even though Mg^2+^ has very weak ability to bundle VIFs in bulk solution, when vimentin filaments are confined in small droplets approximately the size of a cell, they diffuse as single filaments at [Mg^2+^] = 4 mM, but aggregate over a time scale of 100 s at higher concentrations. The critical aggregation concentration in this study was 14.5 mM, which is well below the bundling threshold in bulk solution (Figure 3A) suggesting that spatial confinement might augment the effect of counterions [22]. Similarly, cytoskeletal systems containing F-actin and microtubules in microfluidic systems contract after addition of 20 mM Mg^2+^, and continue to contract when [Mg^2+^] is lowered to 2 mM, the normal concentration in the cell where most F-actin and microtubules are single filaments [23].

### 2.4. Polyelectrolyte Aggregation Efficiency Correlates with Metal Ion Softness But Not Size

**Figure 4:**
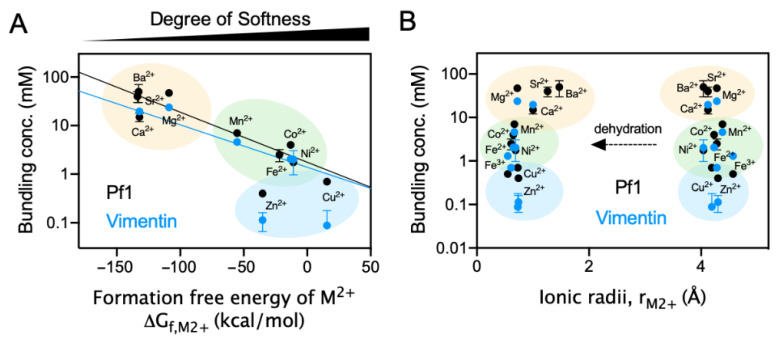
Critical bundling concentrations of different cations for both Pf1 and VIFs correlate with the metal ion softness but not their radii. (**A**). The critical bundling concentrations of different metal ions linearly correlates with the Gibbs free energy of the formation of metal ions, a natural index of Lewis acid softness. (**B**). The trends in the critical bundling concentrations could not be explained by the size effect of the metal ions. The hydrated radii of the metal ions are shown on the right and the naked ionic radii are shown on the left. The beige color on both panels highlights the alkaline earth metals; the light green and blue color highlight the early and late transition metals in the same period, respectively.

The pattern of increasing bundling efficiency among the transition metal ions scales approximately with atomic weight, but the trend among alkaline earth ions does not. Bundling efficiency also does not scale with either the hydrated or dehydrated ion diameter (Figure 4B). Bundling efficiency does, however, appear to be explained by a qualitative hard/soft acid/base theory (HSAB) [24]. The HSAB theory predicts that the stability of a metal–ligand complex increases when a hard–hard or soft–soft interaction between metal and ligand is formed. A mismatch between hard metal and soft ligand or vice versa leads to a decreased complex stability. Hardness or softness of an ion depends on a number of factors including ionic radii, the second ionization potentials of the metal, the interaction between the metal ion and surrounding water, and the steric effect of the metal ion on coordination environments. Cations with a high softness tend not to lose electrons but to share them.

The bundling/crosslinking of polyelectrolyte biopolymers is mediated in part by classical counterion condensation [25] in which counterions like Mg^2+^ are sequestered near the surface of the polyelectrolyte but remain hydrated and free to diffuse along the filament [26], but can be augmented by interactions between anionic amino acid residues on the biopolymers and the divalent metal ions. Because the anionic amino acids that provide the negative surface charge of Pf1 and VIFs are characterized as soft ligands [24], it is predicted that the trend of metal ions in inducing biopolymer bundling increases with increasing softness of the metal ion. This prediction is supported by our data (Figure 4A). The formation free energies of M^2+^ presented in panel A are natural indices for the hardness/softness of metal cations and the softness of metal ions increases along the *x*-axis. This result suggests that the natural hardness/softness of the metal ion and fact that the negative charges on biopolymers are on soft anions provides at least a qualitative interpretation for the order of different divalent cations. The largest discrepancies in the linear fit to the data in Figure 4A are for Zn^2+^ and Cu^2+^ and VIFs. These outlying data points might result from the fact that VIFs also contained cysteines that can bind Zn and Cu, or possibly from phosphorylated amino acids that can occur in VIFs but not Pf1.

## 3. Conclusions

Filamentous polyelectrolytes, as exemplified by vimentin intermediate filaments (VIFs) and Pf1 bacteriophage, form viscoelastic networks and develop attractive interactions in the presence of appropriate concentrations and types of divalent and multivalent counterions. Alkaline earth ions are relatively weak gelators of these filaments, but divalent cations of transition metals are much more efficient, and their efficiency increases with increasing atomic weight. As a result, Cu^2+^ and Zn^2+^ form aggregates of both Pf1 and VIFs at submicromolar concentration, whereas 10 mM Mg^2+^ has no such effect. These results reveal large differences in the ability of metal ions of the same charge to condense filamentous polyelectrolytes and suggest that some of these effects might be relevant in biological settings.

## 4. Materials and Methods

Pf1 phage strain LP11-92 isolated from wild type Pseudomonas aeruginosa and propagated in the phage free strain LA23-99 was obtained from Asla Biotech Ltd. Riga, LV-1067, Latvia. 5 mg/mL solutions of Pf1 virus were made in solutions of 2 mM HEPES, pH 7.5, and 1 mM NaN_3_. Vimentin was obtained from Prof Peter Traub and polymerized in solutions containing 5 mM Tris, 1 mM EGTA, 0.1 mM DTT, 5 mM 2-mercaptoethanol, 150 mM KCI, pH 7.6 [27,28]. Hyaluronic acid was obtained from Sigma Aldrich, Inc (St, Louis, MO) and suspended in phosphate buffered saline. Divalent and multivalent counterions were added from stock solutions of 10 mM to 1 M as needed to increase counterion concentrations without diluting the sample more than 10%. Metal ions supplied as acetates or chlorides depending on higher solubility, were reagent grade. 

Pf1 was visualized by atomic force microscopy using a Veeco Bioscope II instrument (Santa Barbara, CA, USA), using methods previously described to visualize neurofilaments [29]. The viscoelastic properties of Pf1 suspensions were quantified by their shear compliance after imposition of variable levels of shear stress to a sample confined between two parallel plates. Compliance is defined as the ratio of shear strain to shear stress. The shear compliance was determined from the relation between applied force and the resulting deformation using a Bohlin CVO Rheometer (Malvern, Inc. Malvern UK). The initial time-dependent increase in strain is referred to as creep, and the subsequent decrease in strain when the stress is removed is creep recovery. 

Dynamic light scattering (DLS) spectroscopy was used to assess the formation of Pf1, and VIF aggregates in the presence of metal ions. The average aggregate size (apparent hydrodynamic diameter) was determined using a DynaPro 99 DLS instrument (Wyatt Technology Corp. Santa Barbara, CA, USA). The method measures the diffusion constant of the aggregates from the autocorrelation function of scattered light intensity. The apparent size, summarized from the equivalent diffusion constant of a sphere is calculated from the relation D = kT/6πη Rh, where D is the translational diffusion constant, k is Boltzmann’s constant, T is temperature, η is the solvent viscosity, and Rh is the hydrodynamic radius.

## Abbreviations

DLS	dynamic light scattering
HSAB	hard/soft acid/base
PE	polyelectrolyte
PMF	potential of mean force
VIF	vimentin intermediate filament

## References

[1] Janmey P.A., Slochower D.R., Wang Y.H., Wen Q., Cebers A. (2014). Polyelectrolyte properties of filamentous biopolymers and their consequences in biological fluids. Soft Matter.

[2] Hosek M., Tang J.X. (2004). Polymer-induced bundling of F actin and the depletion force. Phys. Rev. E.

[3] Tang J.X., Janmey P.A. (1996). The polyelectrolyte nature of F-actin and the mechanism of actin bundle formation. J. Biol. Chem..

[4] Gurmessa B., Francis M., Rust M.J., Das M., Ross J.L., Robertson-Anderson R.M. (2019). Counterion crossbridges enable robust multiscale elasticity in actin networks. Phys. Rev. Res..

[5] Tang J.X., Wong S.E., Tran P.T., Janmey P.A. (1996). Counterion induced bundle formation of rodlike polyelectrolytes. Ber. Der Bunsen-Ges. Phys. Chem. Chem. Phys..

[6] Bucki R., Durnas B., Watek M., Piktel E., Cruz K., Wolak P., Savage P.B., Janmey P.A. (2018). Targeting polyelectrolyte networks in purulent body fluids to modulate bactericidal properties of some antibiotics. Infect. Drug Resist..

[7] O’Reilly S.A., Roedica J., Nagy D., Hallewell R.A., Alderson K., Marklund S.L., Kuby J., Kushner P.D. (1995). Motor neuron-astrocyte interactions and levels of Cu,Zn superoxide dismutase in sporadic amyotrophic lateral sclerosis. Exp. Neurol..

[8] Yemets A., Horiunova I., Blume Y. (2021). Cadmium, nickel, copper, and zinc influence on microfilament organization in Arabidopsis root cells. Cell Biol. Int..

[9] Herrmann H., Haner M., Brettel M., Muller S.A., Goldie K.N., Fedtke B., Lustig A., Franke W.W., Aebi U. (1996). Structure and assembly properties of the intermediate filament protein vimentin: The role of its head, rod and tail domains. J. Mol. Biol..

[10] Liu D.J., Day L.A. (1994). Pf1 virus structure: Helical coat protein and DNA with paraxial phosphates. Science.

[11] Huisman E.M., Wen Q., Wang Y.H., Cruz K., Kitenbergs G., Erglis K., Zeltins A., Cebers A., Janmey P.A. (2011). Gelation of semiflexible polyelectrolytes by multivalent counterions. Soft Matter.

[12] Horkay F., Basser P.J., Hecht A.M., Geissler E. (2018). Ionic effects in semi-dilute biopolymer solutions: A small angle scattering study. J. Chem. Phys..

[13] Zheng Y.T., Lin C., Zhang J.S., Tan Z.J. (2020). Ion-mediated interactions between like-charged polyelectrolytes with bending flexibility. Sci. Rep..

[14] Apelblat A., Manzurola E. (1999). Solubilities of magnesium, calcium, barium, cobalt, nickel, copper, and zinc acetates in water from T = (278.15 to 348.15) K. J. Chem. Thermodyn..

[15] Tang J.X., Janmey P.A., Lyubartsev A., Nordenskiold L. (2002). Metal ion-induced lateral aggregation of filamentous viruses fd and M13. Biophys. J..

[16] Slochower D.R., Huwe P.J., Radhakrishnan R., Janmey P.A. (2013). Quantum and all-atom molecular dynamics simulations of protonation and divalent ion binding to phosphatidylinositol 4,5-bisphosphate (PIP2). J. Phys. Chem. B.

[17] Perez-Sala D., Oeste C.L., Martinez A.E., Carrasco M.J., Garzon B., Canada F.J. (2015). Vimentin filament organization and stress sensing depend on its single cysteine residue and zinc binding. Nat. Commun..

[18] Monico A., Zorrilla S., Rivas G., Perez-Sala D. (2020). Zinc Differentially Modulates the Assembly of Soluble and Polymerized Vimentin. Int. J. Mol. Sci..

[19] Wu H.Y., Shen Y.N., Wang D.Z., Herrmann H., Goldman R.D., Weitz D.A. (2020). Effect of Divalent Cations on the Structure and Mechanics of Vimentin Intermediate Filaments. Biophys. J..

[20] Li S., Zhang J., Yang H., Wu C., Dang X., Liu Y. (2015). Copper depletion inhibits CoCl2-induced aggressive phenotype of MCF-7 cells via downregulation of HIF-1 and inhibition of Snail/Twist-mediated epithelial-mesenchymal transition. Sci. Rep..

[21] Zhu P., Hawkins J., Linthicum W.H., Wang M., Li N., Zhou N., Wen Q., Timme-Laragy A., Song X., Sun Y. (2020). Heavy Metal Exposure Leads to Rapid Changes in Cellular Biophysical Properties. ACS Biomater. Sci. Eng..

[22] Dammann C., Koster S. (2014). Dynamics of counterion-induced attraction between vimentin filaments followed in microfluidic drops. Lab Chip.

[23] Ricketts S.N., Khanal P., Rust M.J., Das M., Ross J.L., Robertson-Anderson R.M. (2020). Triggering Cation-Induced Contraction of Cytoskeleton Networks via Microfluidics. Front. Phys..

[24] Xu H., Xu D.C., Wang Y. (2017). Natural Indices for the Chemical Hardness/Softness of Metal Cations and Ligands. ACS Omega.

[25] Manning G.S., Ray J. (1998). Counterion condensation revisited. J. Biomol. Struct. Dyn..

[26] Xian W., Tang J.X., Janmey P.A., Braunlin W.H. (1999). The polyelectrolyte behavior of actin filaments: A 25Mg NMR study. Biochemistry.

[27] Janmey P.A., Euteneuer U., Traub P., Schliwa M. (1991). Viscoelastic properties of vimentin compared with other filamentous biopolymer networks. J. Cell Biol..

[28] Tang J.X., Ito T., Tao T., Traub P., Janmey P.A. (1997). Opposite effects of electrostatics and steric exclusion on bundle formation by F-actin and other filamentous polyelectrolytes. Biochemistry.

[29] Wagner O.I., Lifshitz J., Janmey P.A., Linden M., McIntosh T.K., Leterrier J.F. (2003). Mechanisms of mitochondria-neurofilament interactions. J. Neurosci..

